# Nanotechnology for brain tumor imaging and therapy based on π-conjugated materials: state-of-the-art advances and prospects

**DOI:** 10.3389/fchem.2023.1301496

**Published:** 2023-11-08

**Authors:** Wenshe Sun, Congxiao Wang, Chuan Tian, Xueda Li, Xiaokun Hu, Shifeng Liu

**Affiliations:** ^1^ Department of Interventional Medical Center, Affiliated Hospital of Qingdao University, Qingdao, Shandong, China; ^2^ Qingdao Cancer Institute, Qingdao University, Qingdao, China

**Keywords:** π-conjugated materials, brain tumor, molecular design, fluorescence imaging, photothermal therapy, photodynamic therapy

## Abstract

In contemporary biomedical research, the development of nanotechnology has brought forth numerous possibilities for brain tumor imaging and therapy. Among these, π-conjugated materials have garnered significant attention as a special class of nanomaterials in brain tumor-related studies. With their excellent optical and electronic properties, π-conjugated materials can be tailored in structure and nature to facilitate applications in multimodal imaging, nano-drug delivery, photothermal therapy, and other related fields. This review focuses on presenting the cutting-edge advances and application prospects of π-conjugated materials in brain tumor imaging and therapeutic nanotechnology.

## 1 Introduction

π-Conjugated materials, a class of organic molecules or polymers with conjugated structures, possess extensive potential applications in the field of biomedicine (Pimachev et al., 2019). Their unique electronic structure and optical properties make them ideal candidates for biomedical imaging and therapy (Ma et al., 2021; Deng et al., 2022). By altering their conjugated structure and side-chain functional groups, π-conjugated materials can modulate absorption and fluorescence emission peaks, enabling high-selectivity imaging of biological tissues. Additionally, due to their excellent photothermal conversion performance, they can be utilized in photothermal therapy, generating localized temperature elevation through light energy conversion to deactivate tumor cells (Zhou and Liang, 2014; Parida et al., 2022). Consequently, the application of π-conjugated materials in biomedicine has become a focal point of research.

While π-conjugated materials have made significant strides in brain tumor imaging and therapy, their design, preparation, and clinical application still face certain limitations and challenges. To delineate the primary issues and unresolved matters concerning π-conjugated materials in these aspects, it is imperative to systematically review the existing research frontier to guide the direction of future studies. This paper aims to provide a thorough analysis and comparison of relevant research findings to clearly delineate the major knowledge gaps and limitations of π-conjugated materials in the following areas, with the intention of steering future research efforts:

Firstly, it will introduce the applications of π-conjugated materials in brain tumor imaging, including the design and preparation methods of various π-conjugated nanoprobes and their usage in multimodal imaging such as magnetic resonance imaging (MRI), fluorescence imaging, and photoacoustic imaging. By conducting comparative analyses of different imaging modalities, the advantages and challenges of π-conjugated materials in brain tumor imaging will be explored.

Secondly, this review will delve into the applications of π-conjugated materials in brain tumor therapy, with a particular focus on research progress in nanodrug delivery systems, photothermal therapy, and photodynamic therapy. Through a comprehensive review of the advantages and disadvantages of different therapeutic strategies, the potential value of π-conjugated materials in brain tumor treatment will be discussed. Moreover, the review will also discuss the application prospects of π-conjugated materials in combination therapy, exploring the synergistic effects of various treatment strategies and providing new insights and directions for precision treatment of brain tumors.

Lastly, this review will critically analyze the biological safety and toxicity assessment of π-conjugated materials in brain tumor therapy. By reviewing relevant literature, the metabolic pathways and biodistribution of π-conjugated materials *in vivo*, as well as their interactions with normal tissues and organs, will be comprehensively evaluated. A thorough exploration of the biocompatibility and potential toxicity of π-conjugated materials will be conducted, providing important references for further clinical applications.

**FIGURE 1:**
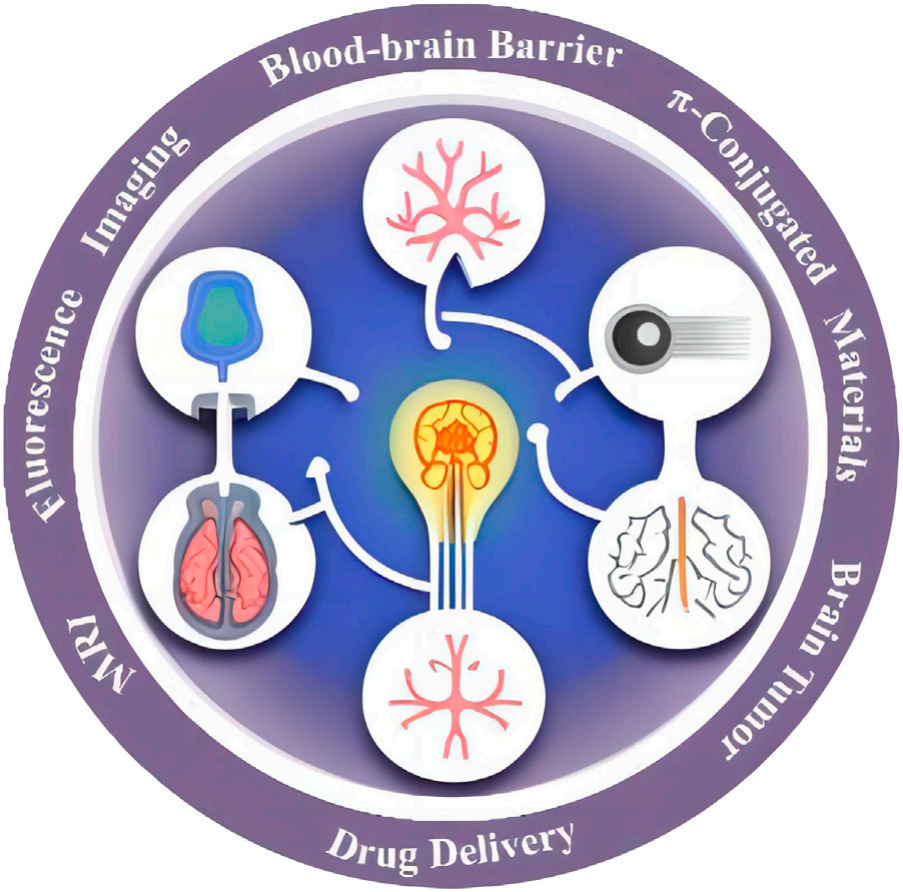
Potential applications of π conjugate materials in brain tumors.

Through a comprehensive review of the applications of π-conjugated materials in brain tumor imaging and therapeutic nanotechnology, we aim to deepen our understanding of this field, promote the translational application of π-conjugated materials in clinical brain tumor treatment, and provide patients with more precise and effective therapeutic options (Figure 1).

## 2 Application of π-conjugated materials in brain tumor imaging

### 2.1 Design and preparation of π-conjugated nanoprobes

**FIGURE 2:**
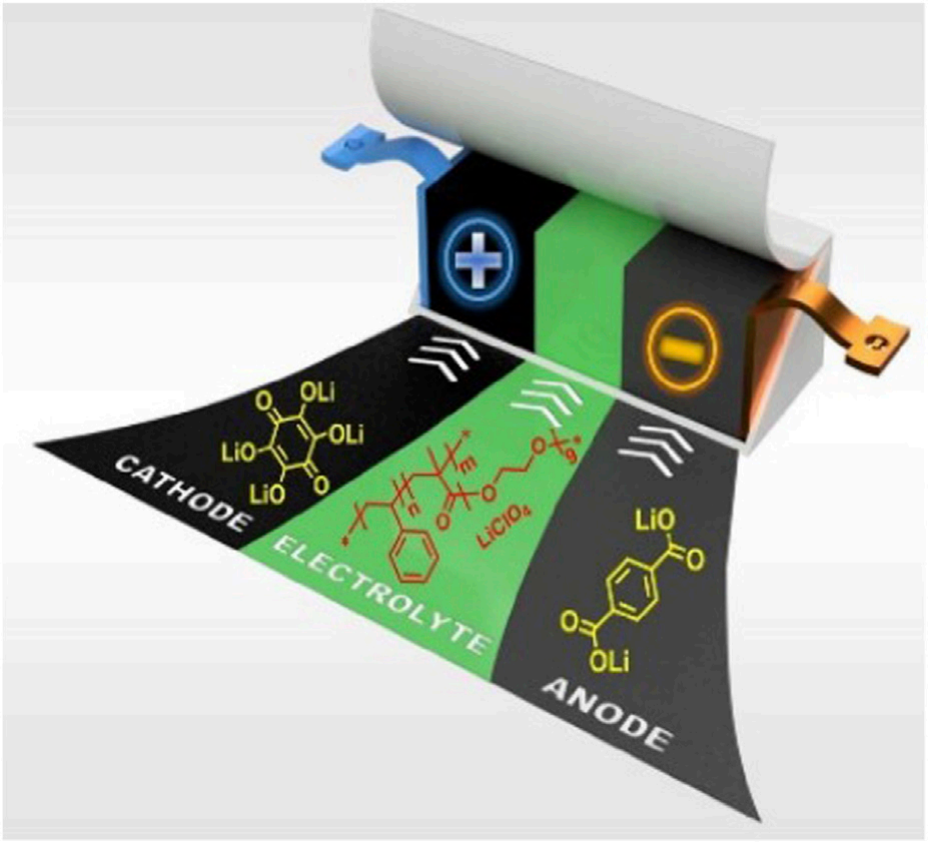
π-conjugated materials: from synthesis to applications (Oubaha et al., 2019).

π-Conjugated materials, as a unique class of nanoprobes, hold broad prospects for brain tumor imaging (Hiroto and Wu, 2019; Li et al., 2021a). In this section, we will focus on the design and preparation methods of π-conjugated nanoprobes, emphasizing their advantages and application value in brain tumor imaging. Firstly, material selection is crucial in the design of π-conjugated nanoprobes (Li et al., 2023). Generally, the materials for π-conjugated nanoprobes should possess excellent optical properties and biocompatibility (Hiroto and Wu, 2019). Commonly used materials include metallic nanoparticles, carbon nanotubes, and quantum dots. Metallic nanoparticles exhibit outstanding surface plasmon resonance effects, enhancing fluorescence signals (Haque et al., 2020); carbon nanotubes possess excellent optical properties and mechanical strength for biomedical imaging and drug delivery; quantum dots offer tunable size and narrow emission spectra, suitable for multi-channel imaging (Miki and Ohe, 2020; Lao et al., 2022). Secondly, the synthesis method is a critical step in the preparation of π-conjugated nanoprobes. Common synthesis methods include solution-based, gas-phase, and solid-phase approaches (Yoon and Dong, 2021). The solution-based method is one of the most commonly used methods, achieving controlled synthesis of nanomaterials through adjusting reaction conditions and adding surfactants (Hicks et al., 2021; M et al., 2022). The gas-phase method involves converting gaseous precursors into nanoparticles through thermal evaporation or pyrolysis (Figure 2). The solid-phase method converts solid precursors into nanomaterials through thermal treatment (Oubaha et al., 2019).

**FIGURE 3:**
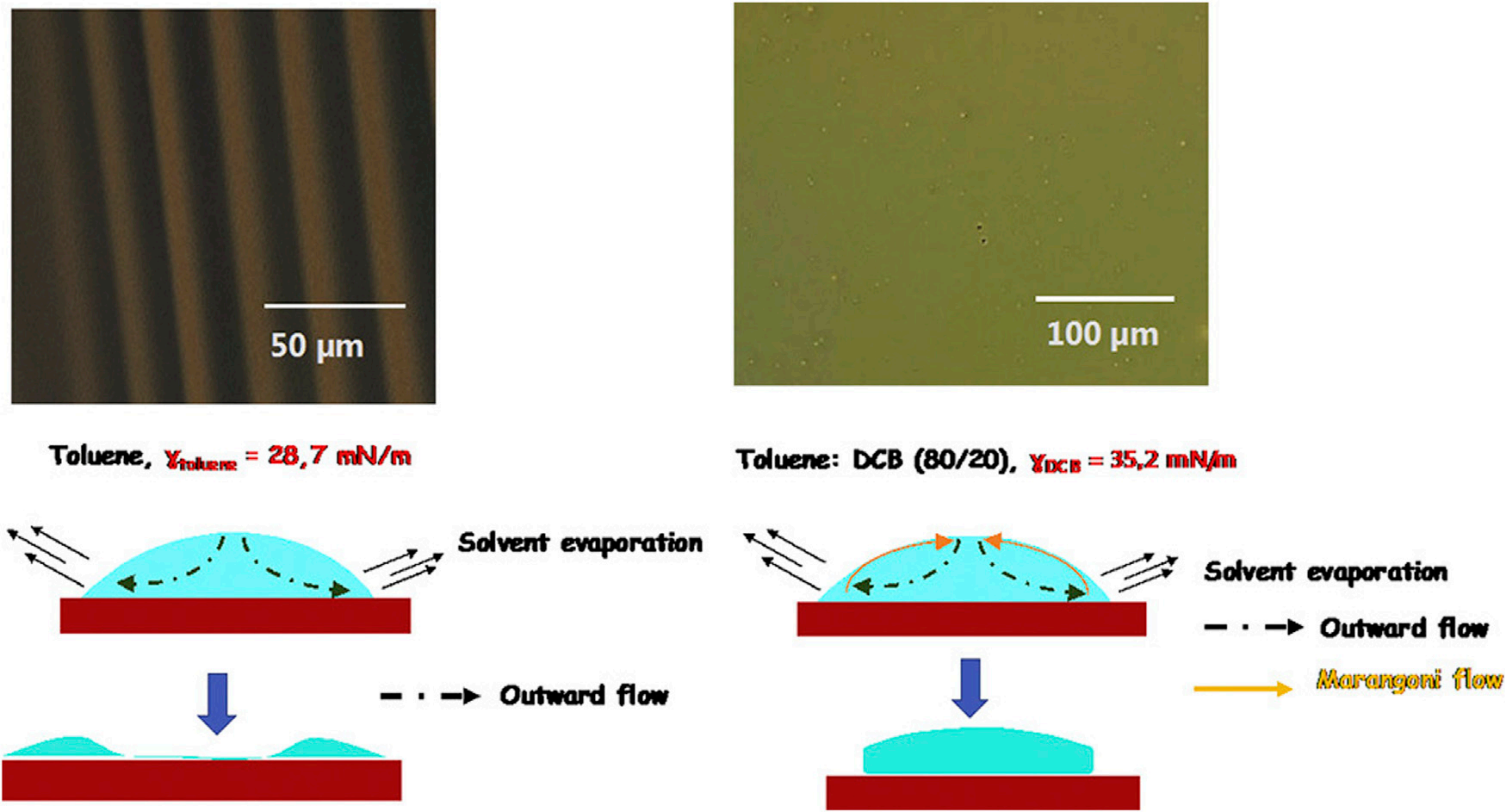
Large-scale patterning of π-conjugated materials (Richard et al., 2020).

Common characterization techniques include transmission electron microscopy (TEM), scanning electron microscopy (SEM), UV-Vis absorption spectroscopy, and fluorescence spectroscopy (Figure 3). TEM and SEM are used to observe the morphology and size distribution of nanoprobes; UV-Vis absorption spectroscopy characterizes their optical properties; fluorescence spectroscopy evaluates the fluorescence intensity and emission spectra of nanoprobes (Naciri et al., 2020; Zhao, 2020; Haque et al., 2023). By selecting appropriate materials, optimizing synthesis methods, and accurately characterizing performance, π-conjugated nanoprobes with excellent properties can be fabricated, providing strong support for research in areas such as biomedical imaging and drug delivery (Xu et al., 2015; Li and Pu, 2019; Song et al., 2022).

In the practical application of brain tumors, the key to designing and preparing π-conjugated nanoprobes lies in the modulation of their structure and properties to achieve highly selective imaging of brain tumors (Stahl et al., 2017; Yin et al., 2017). Firstly, researchers typically design suitable targeting ligands, such as antibodies, oligonucleotides, and peptides, based on the specific surface biomarkers of brain tumor cells, and then modify them on the surface of π-conjugated materials (Guo et al., 2018). Such targeting modification can improve the nanoprobes’ cell recognition and affinity, achieving more accurate imaging of brain tumors (Jiang et al., 2019; Li and Pu, 2019; Neumann et al., 2021). Secondly, adjusting the physical properties of π-conjugated materials, such as tuning the fluorescence emission peak and altering the absorption spectra, enables the selection of different imaging modes (Kubo, 2019; Ong et al., 2022). For instance, fluorescence emission peaks in the near-infrared region can reduce interference from tissue autofluorescence, enhancing imaging depth and signal-to-noise ratio. By designing the size and surface modification of nanoprobes rationally, penetration through the blood-brain barrier can be achieved, enhancing the targeting of brain tumors (Samori et al., 2019). Furthermore, the stability and biocompatibility of nanoprobes are also important factors to consider in the design process (Zhang et al., 2016). Through appropriate surface modification and coating materials, the stability of nanoprobes can be enhanced, extending their circulation time in the body. Simultaneously, studying the metabolic pathways and biodistribution of nanoprobes in the body contributes to assessing their biocompatibility and safety (Montalti et al., 2015).

In conclusion, the design and preparation of π-conjugated nanoprobes involve interdisciplinary collaboration, requiring in-depth research in nanomaterials, biomedical sciences, and chemistry (Xu et al., 2015). Through careful design and rational preparation, π-conjugated nanoprobes possess high targeting specificity and biocompatibility in brain tumor imaging, providing new means and tools for early detection and quantitative analysis of brain tumors (Kaeser and Schenning, 2010; Lin et al., 2022).

### 2.2 Application of π-conjugated materials in multimodal imaging

**FIGURE 4:**
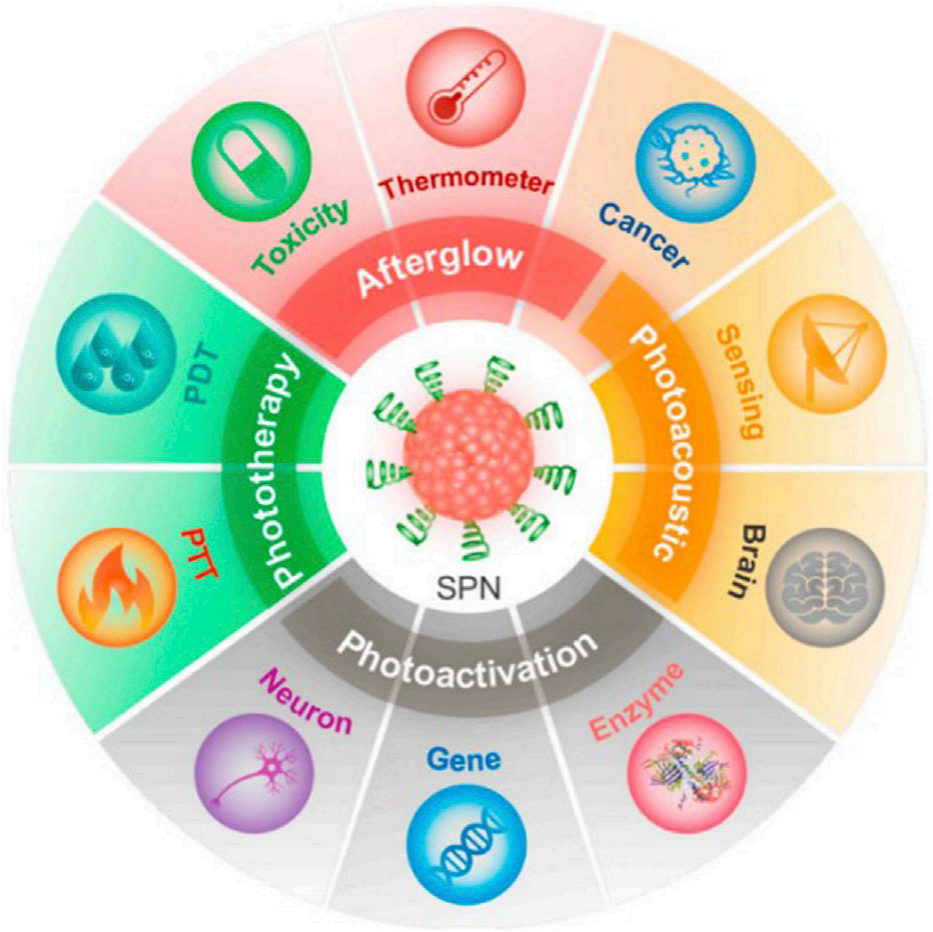
Schematic representation molecules developed for conjugated materials and photophysical processes.

The application of π-conjugated materials in multimodal imaging is currently a hot topic in brain tumor research (Ge et al., 2023). Multimodal imaging techniques integrate different imaging modalities organically, providing more comprehensive and accurate information on brain tumors, thereby offering strong support for clinical diagnosis and treatment decisions (Gu et al., 2015; Li S. et al., 2022). In this section, we will explore the application of π-conjugated materials in multimodal imaging, as well as their advantages and limitations in brain tumor imaging (Figure 4).

π-Conjugated materials are a class of organic materials with unique electronic structures, and their electrons can freely move within the molecular backbone, forming a π-electron cloud (Mirzaei et al., 2021). This characteristic confers excellent optical properties on π-conjugated materials, including broad absorption and emission spectra and high-efficiency light conversion (Yin et al., 2021). These advantages endow π-conjugated materials with extensive applications in multimodal imaging (Zhan and Liu, 2016).

#### 2.2.1 Magnetic resonance imaging (MRI)

The utilization of π-conjugated materials in MRI plays a pivotal role in multimodal imaging. Through surface modifications of π-conjugated materials on magnetic nanoparticles, a substantial enhancement in MRI contrast for brain tumors has been achieved, enabling high-resolution imaging. Furthermore, the tuning of their electronic structure and magnetic properties has demonstrated the potential to modulate their magnetic resonance signals, contributing to improved contrast in MRI (Zhu et al., 2018). Additionally, the targeted modification of π-conjugated nanoprobes has displayed the capacity to enhance their specificity for brain tumors, thereby augmenting the sensitivity of MRI imaging (Schmitt et al., 2018).

#### 2.2.2 Fluorescence imaging

The outstanding fluorescence properties of π-conjugated materials have garnered significant attention in brain tumor fluorescence imaging (Doan et al., 2022). Structural modulation enables the adjustment of the fluorescence emission peak of π-conjugated materials, extending the emission into the near-infrared region to enhance imaging depth and signal-to-noise ratio (Chatterjee et al., 2020; Irshad et al., 2023). Moreover, in conjunction with targeted modifications, π-conjugated nanoprobes have achieved highly selective brain tumor imaging, offering pivotal insights for precise diagnosis. Several π-conjugated polymers and small molecules have been effectively employed in in vivo imaging, demonstrating remarkable biocompatibility and efficient fluorescence emission (Liu et al., 2013; Chen et al., 2022).

#### 2.2.3 Photoacoustic imaging

An emerging biological imaging technique, photoacoustic imaging, has demonstrated the ability to achieve high-contrast imaging of brain tumors through photothermal effects. The exceptional photothermal conversion performance of π-conjugated materials positions them as ideal probes for photoacoustic imaging (Yao et al., 2022). In this context, π-conjugated nanoprobes can generate acoustic signals through laser-induced photothermal effects, enabling three-dimensional brain tumor imaging and presenting a novel avenue for non-invasive detection of brain tumors (Neumann et al., 2021). Some π-conjugated polymers have already proven to be efficient photoacoustic contrast agents for imaging tumors and blood vessels (Stahl et al., 2017).

#### 2.2.4 Photothermal imaging

**FIGURE 5:**
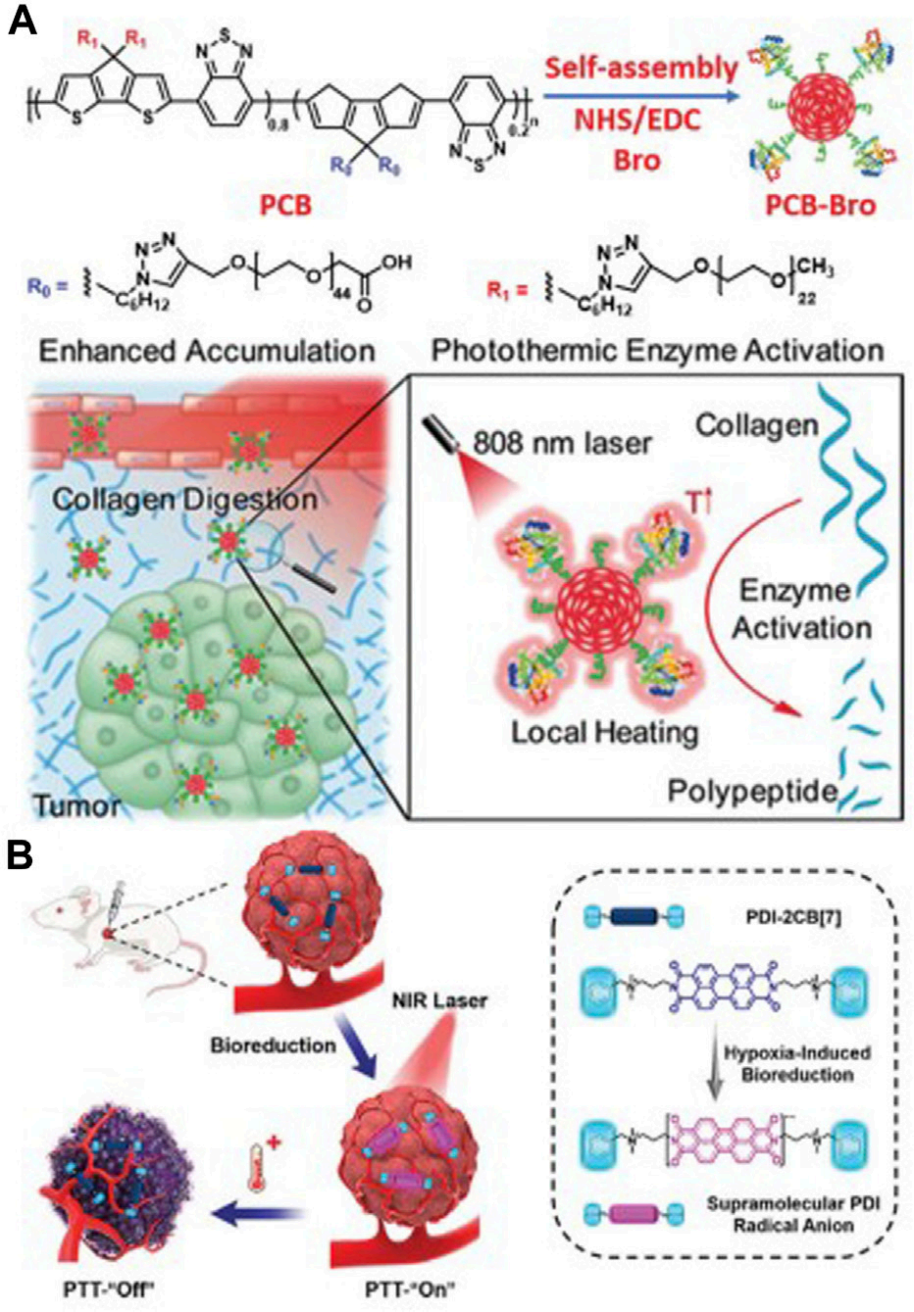
**(A)** The preparation process of PCB-Bro and its role in facilitating collagen digestion to enhance its tumor accumulation are depicted herein. **(B)** A schematic representation elucidates the generation of supramolecular PDI radical anions within tumors, serving as a specific mechanism for photothermal therapy (PTT). Adapted with permission from Lin et al. (2022).

The photothermal conversion efficiency of π-conjugated materials has garnered extensive attention (Figure 5), and certain π-conjugated materials have demonstrated their efficiency as photothermal conversion agents for photothermal imaging and therapy (Chen et al., 2022; Yao et al., 2022).

### 2.3 Performance optimization and future development of imaging techniques

As the application of π-conjugated materials in brain tumor imaging becomes increasingly widespread, the optimization and development of imaging techniques become particularly important. The performance of imaging techniques directly affects the accuracy of brain tumor detection and quantitative analysis (Chen et al., 2004). In this section, we will discuss key technical optimization measures and future development directions to improve the application effectiveness of π-conjugated materials in brain tumor imaging.

#### 2.3.1 Magnetic resonance imaging (MRI) optimization

MRI is a powerful non-invasive imaging technique widely used for diagnosing and monitoring various diseases, including brain tumors (Villanueva-Meyer et al., 2017). While MRI enables precise diagnosis without invasive procedures, there is still room for significant improvement in the quality and accuracy of the images produced. To enhance MRI contrast and sensitivity for brain tumor imaging using π-conjugated materials modified magnetic nanoparticles, further optimization of their magnetic properties is necessary (Soffietti et al., 2020; Ranjbarzadeh et al., 2023). The size, shape, and surface modifications of magnetic nanoparticles play a crucial role in their imaging performance in MRI. Therefore, optimizing the physical properties of the nanoprobes is essential to achieve high-resolution imaging of brain tumors.

The research “Application of π-Conjugated Materials in MRI-Guided Brain Tumor Diagnosis and Treatment” discusses the impact of π-conjugated materials as MRI contrast agents on brain tumor diagnosis and treatment (Smits, 2021). The study shows that due to the excellent optical and electronic properties of π-conjugated materials, they can generate exceptional contrast in MRI, allowing physicians to clearly visualize the size, shape, and location of brain tumors. Moreover, their characteristics enable them to maintain stability under high magnetic field strength, optimizing the accuracy of MRI scans (Brindle et al., 2017). These factors help doctors develop more precise treatment strategies, thus improving patient outcomes. Another research paper titled “Optimizing MRI Scans: Enhancing Brain Tumor Imaging Quality Using π-Conjugated Materials” aims to improve brain tumor imaging quality by optimizing MRI protocols and techniques and using specific types and quantities of π-conjugated materials (van der Voort et al., 2023). The researchers explore various methods and parameter adjustments, such as magnetic field strength, scanning time, and the type and amount of π-conjugated materials used, to determine which factors significantly enhance imaging quality (Ellingson et al., 2015). The results indicate that using specific types and quantities of π-conjugated materials during MRI scanning can substantially improve image clarity and resolution, allowing physicians to more accurately identify the tumor’s location and size (Kim et al., 2019). The article titled “The Importance of Using π-Conjugated Materials in MRI for Early Detection of Brain Tumors” discusses the value of π-conjugated materials in early brain tumor detection using MRI. This article extensively explains how the high contrast provided by π-conjugated materials in MRI imaging allows doctors to detect tumors even in their early stages (Bangalore Yogananda et al., 2020). The exceptional contrast enables physicians to observe subtle structural changes, providing higher sensitivity and specificity than traditional MRI techniques (Peng et al., 2022). Early detection allows doctors to begin treatment before the tumor progresses to more severe stages, significantly improving patient survival rates.

In summary, by using π-conjugated materials and optimizing MRI techniques and protocols, we can greatly enhance the effectiveness and accuracy of brain tumor imaging, providing physicians with more precise information to develop better treatment strategies. Through these advancements, medical imaging capabilities in diagnosing and treating brain tumors have significantly improved. However, further research is needed to fully understand all potential applications of π-conjugated materials in MRI optimization and determine their optimal use.

#### 2.3.2 Fluorescence imaging optimization

In π-conjugated materials’ fluorescence imaging, optimizing the optical properties is essential. Adjusting the structure and functional groups of π-conjugated materials to tune the fluorescence emission peak to the near-infrared range can reduce interference from tissue autofluorescence, improving the imaging signal-to-noise ratio. Additionally, enhancing the fluorescence quantum yield and stability of the nanoprobes can enable long-term real-time monitoring.

The research titled “Optimization of Brain Tumor Imaging using Self-Assembled Nano Fluorescent Probes” utilizes self-assembled nano fluorescent probes for imaging brain tumors in mice (Wen et al., 2019). By optimizing the probes’ fluorescence characteristics and optical properties, the research team achieved highly sensitive brain tumor imaging. The results showed that the self-assembled nano fluorescent probes exhibited a comparative advantage in brain tumor imaging while minimally affecting surrounding normal tissues (Li et al., 2019a; Wen et al., 2019). Another research paper, “Optimization of Brain Tumor Imaging using Multi-Modal Fluorescent Probes,” employed a multi-modal fluorescent probe that combined different fluorescence imaging modes, such as fluorescence resonance energy transfer and fluorescence excitation spectroscopy, to enhance the accuracy and resolution of brain tumor imaging (Tomitaka et al., 2019). The results demonstrated that the multi-modal fluorescent probe offered high sensitivity and specificity in brain tumor imaging, effectively locating and identifying brain tumor tissue (Tomitaka et al., 2019). Additionally, a study titled “Enhancing the Application of Fluorescent Conjugated Materials in Brain Tumor Imaging” focused on optimizing the application of fluorescent conjugated materials in brain tumor imaging (Sheikh Mohamed et al., 2016). The research team designed and synthesized a series of fluorescent conjugated materials with different structures and properties, validating their performance in brain tumor imaging through animal experiments (Sheikh Mohamed et al., 2016). The results showed that the optimized fluorescent conjugated materials had higher fluorescence brightness, stability, and biocompatibility, enabling high-resolution and high-contrast brain tumor imaging (Sheikh Mohamed et al., 2016).

In conclusion, optimizing the application of π-conjugated materials in fluorescence imaging for brain tumors is a promising research field. These studies provide crucial support for early detection and accurate diagnosis of brain tumors, laying the foundation for subsequent research and clinical applications. With ongoing technological development and innovation, more breakthroughs are expected in enhancing the application of π-conjugated materials in brain tumor imaging.

#### 2.3.3 Photoacoustic imaging optimization

In photoacoustic imaging, it is essential to optimize the photoacoustic conversion efficiency of π-conjugated nanoprobes and the sensitivity of acoustic signal detection (Wu et al., 2022). By carefully selecting the composition and morphology of π-conjugated materials, optimizing photoacoustic effects, and improving the efficiency of acoustic signal detection, researchers can achieve more accurate brain tumor imaging (Guo et al., 2023).

The study “Multi-Modal Photoacoustic and MRI Imaging Detection and Monitoring of Brain Tumors using π-Conjugated Polymer Nanoparticles” combines π-conjugated polymer nanoparticles with photoacoustic and MRI imaging techniques to enhance the sensitivity and resolution of brain tumor imaging (Stahl et al., 2017). The experimental results demonstrate that this multi-modal imaging approach allows high-resolution image reconstruction in deep tissues, facilitating early diagnosis and treatment monitoring. Additionally, the research “Transparency Imaging of Brain Tumors using Photoacoustic Microscopy and π-Conjugated Polymer Nanoparticles” combines photoacoustic microscopy with π-conjugated polymer nanoparticles to achieve transparent imaging of brain tumors (Guo et al., 2018). Through this method, researchers can observe fine internal structures and vascular distribution within brain tumors, furthering the understanding of tumor growth and metastasis mechanisms. This research provides essential groundwork for brain tumor microsurgery and treatment (Guo et al., 2018). Lastly, “Application of π-Conjugated Polymer Nanoprobes in Photoacoustic/MRI Multi-Modal Imaging for Brain Tumor Detection” explores the use of a novel π-conjugated polymer nanoscale probe for multi-modal photoacoustic/MRI imaging in brain tumor detection (Wang et al., 2021). The research team optimized the application of this multi-modal imaging method for tumor localization and edge identification, showing high accuracy, and effectively enhancing treatment planning precision and surgical success rates (Wang et al., 2021).

In summary, optimizing the application of π-conjugated materials through photoacoustic imaging is a cutting-edge research direction. By appropriately designing and improving the properties of π-conjugated materials and combining them with photoacoustic imaging techniques, researchers can enhance brain tumor imaging’s sensitivity, resolution, and accuracy, providing crucial support for early detection and precise treatment of brain tumors. Future research can further explore the combined application of photoacoustic imaging techniques with other medical imaging modalities, offering more possibilities for comprehensive evaluation and accurate treatment of brain tumors.

#### 2.3.4 Multimodal imaging technology integration

The future development will focus on the integration of multimodal imaging technology. The application of π-conjugated materials in various imaging modes provides a strong foundation for achieving multimodal imaging (Lewis et al., 2021). By organically combining different imaging modes, a more comprehensive and accurate depiction of brain tumors can be achieved, enhancing the reliability of diagnosis. The fusion of multimodal imaging technology also allows for complementary information acquisition about brain tumors, aiding in a deeper understanding of their biological characteristics and treatment responses (Cheng et al., 2020).

In conclusion, the application of π-conjugated materials in brain tumor imaging faces numerous opportunities for optimization and development. By appropriately designing the structure and properties of nanoprobes, optimizing the performance of imaging techniques, and achieving the integration of multimodal imaging technology, the application of π-conjugated materials in brain tumor imaging can be enhanced, providing new opportunities and possibilities for early detection and treatment of brain tumors. Future research efforts will promote the extensive application of π-conjugated materials in clinical brain tumor diagnosis and treatment, leading to better clinical outcomes and quality of life for patients (Chen et al., 2014; Neumann et al., 2020).

Wang et al. (2016) reviews the recent progress in the application of multimodal imaging technology using π-conjugated materials for brain tumor imaging. The research finds that by integrating MRI, PET, and optical imaging technologies, the sensitivity and specificity of brain tumor imaging can be improved, providing more accurate information for clinical diagnosis and treatment. Lin et al. (2022) used MRI, PET, and optical imaging technologies to inject π-conjugated materials into a mouse brain tumor model, obtaining accurate brain tumor images through the fusion of multimodal imaging technology. The results indicate that π-conjugated materials have potential application value in brain tumor imaging. Liu et al. (2019) used MRI, PET, and ultrasound imaging technologies to introduce π-conjugated materials into a mouse brain tumor model, obtaining high-resolution brain tumor images through the fusion of multimodal imaging technology. The results show that multimodal imaging technology fusion can improve the accuracy and visualization of brain tumor imaging. A study reviews the prospects of the application of π-conjugated materials in brain tumor imaging and explores the development trends of multimodal imaging technology in this field. The research finds that by integrating MRI, PET, and optical imaging technologies, the accuracy and visualization of brain tumor imaging can be improved, offering new ideas for individualized treatment of brain tumors (Calhoun and Sui, 2016).

In summary, the improvement of the application of multimodal imaging technology in brain tumor imaging is a highly researched area. By integrating MRI, PET, optical imaging, and ultrasound imaging technologies, more accurate and comprehensive brain tumor images can be obtained, providing more information for clinical diagnosis and treatment (Yoon et al., 2014). Future research should further explore the application of multimodal imaging technology using π-conjugated materials to enhance early detection and personalized treatment of brain tumors.

## 3 Application of π-conjugated materials in brain tumor therapy

### 3.1 Advances in nanomedicine delivery systems

Nanomedicine delivery systems involve the nanoscale formulation of drug carriers to achieve precise drug delivery and release, making it a significant research direction in brain tumor therapy (Chen et al., 2022; Narmani et al., 2023). In the context of π-conjugated materials, they serve as excellent nanocarriers, offering new ideas and means for the research of nanomedicine delivery systems.

The advantages of π-conjugated materials lie in their large surface area, tunable optical properties, and good biocompatibility (Li et al., 2022b). These characteristics make them effective drug carriers that can achieve targeted drug delivery through surface modification. By encapsulating drugs within π-conjugated material nanoparticles, the biological distribution of drugs can be improved, effectively reducing toxicity and side effects in normal tissues (Haque et al., 2020). Furthermore, nanomedicine delivery systems can achieve high selective delivery to brain tumor tissues through passive or active targeting strategies, enhancing the local therapeutic effect of drugs (Zhu et al., 2018; Hiroto and Wu, 2019; Haque et al., 2023).

In nanomedicine delivery systems, π-conjugated materials can also enable light-triggered drug release through their optical properties (Xu et al., 2015; Tang et al., 2021). By utilizing the absorption characteristics of π-conjugated materials, drug-loaded nanoparticles can be selectively stimulated to release drugs in specific tumor areas upon exposure to light. This light-triggered drug release approach can significantly reduce nonspecific drug release in the body, enhancing drug targeting and therapeutic efficacy (Qiao et al., 2022).

However, the application of nanomedicine delivery systems also faces some challenges. For instance, the blood-brain barrier restricts drug delivery to brain tissues (Patra et al., 2018). Thus, further optimization and improvement of π-conjugated materials as nanocarriers are needed to overcome this issue. Additionally, comprehensive studies on the biocompatibility and toxicity assessment of π-conjugated materials are necessary to ensure their safety in clinical applications (Haque et al., 2023).

### 3.2 Photothermal therapy and photodynamic therapy

Photothermal therapy and photodynamic therapy are therapeutic approaches that utilize optical properties, enabled by the photothermal conversion performance of π-conjugated materials, for brain tumor treatment. In both therapies, the local photothermal effect of π-conjugated materials plays a central role in the treatment mechanism (Chen et al., 2020).

**FIGURE 6:**
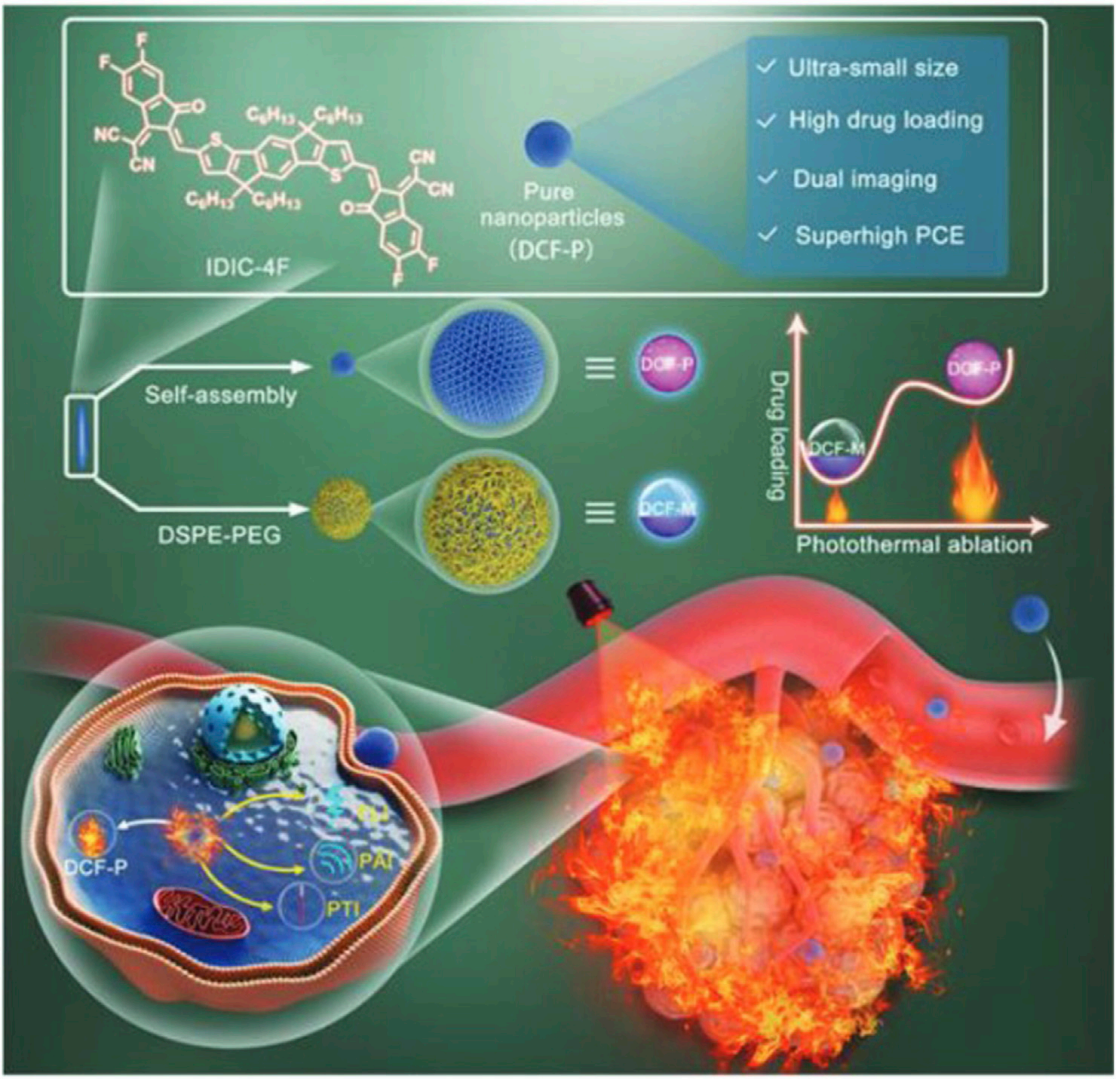
Schematic illustration for carrier-free and carrier-assistant PTT (Zheng et al., 2022).

Photothermal therapy involves exciting the optical properties of π-conjugated materials to convert light energy into heat, generating high temperatures in localized areas with nanoparticles (Guo et al., 2018). This local hyperthermia can lead to the coagulative necrosis of brain tumor cells, achieving tumor ablation. Photothermal therapy offers advantages such as non-invasiveness, excellent local efficacy, and repeatability (Figure 6). By selecting appropriate light parameters and characteristics of π-conjugated materials, efficient brain tumor treatment can be achieved (Xu et al., 2015).

Researchers have synthesized a novel π-conjugated polymer nanoparticle with excellent optical properties, which was used in photothermal therapy for glioma. Experimental results showed that these nanoparticles could generate high temperatures under near-infrared light irradiation, leading to the destruction of brain tumor tissue. This study provides a new method for photothermal therapy of brain tumors. Additionally, π-conjugated carbon dots as optical absorption materials for photothermal therapy and applied them to brain tumor treatment. The experimental results demonstrated that these carbon dots could generate high temperatures under near-infrared light irradiation and accurately locate brain tumor tissue. This research offers a new strategy for photothermal therapy of brain tumors (He et al., 2023). Moreover, a near-infrared phosphorescent π-conjugated metal-organic framework and utilized it in photothermal therapy for brain tumors. The results indicated that this metal-organic framework could generate high temperatures under near-infrared light irradiation and effectively kill brain tumor cells. This study provides a new option for photothermal therapy of brain tumors (Jouaiti et al., 2023). Additionally, a photothermal π-conjugated nanomaterial with excellent optical properties and investigated its application in brain tumor treatment. Experimental results showed that this nanomaterial could generate high heat under light irradiation and effectively kill brain tumor cells. This research offers new perspectives for photothermal therapy of brain tumors (Li et al., 2019b).

In summary, the application of π-conjugated materials in photothermal therapy for brain tumors holds tremendous potential. These studies provide new methods and strategies for photothermal therapy of brain tumors, with the potential to further improve cancer treatment outcomes. Future research can further explore the potential of these π-conjugated materials in clinical applications and accelerate their translation into clinical use (Zheng et al., 2022).

**FIGURE 7:**
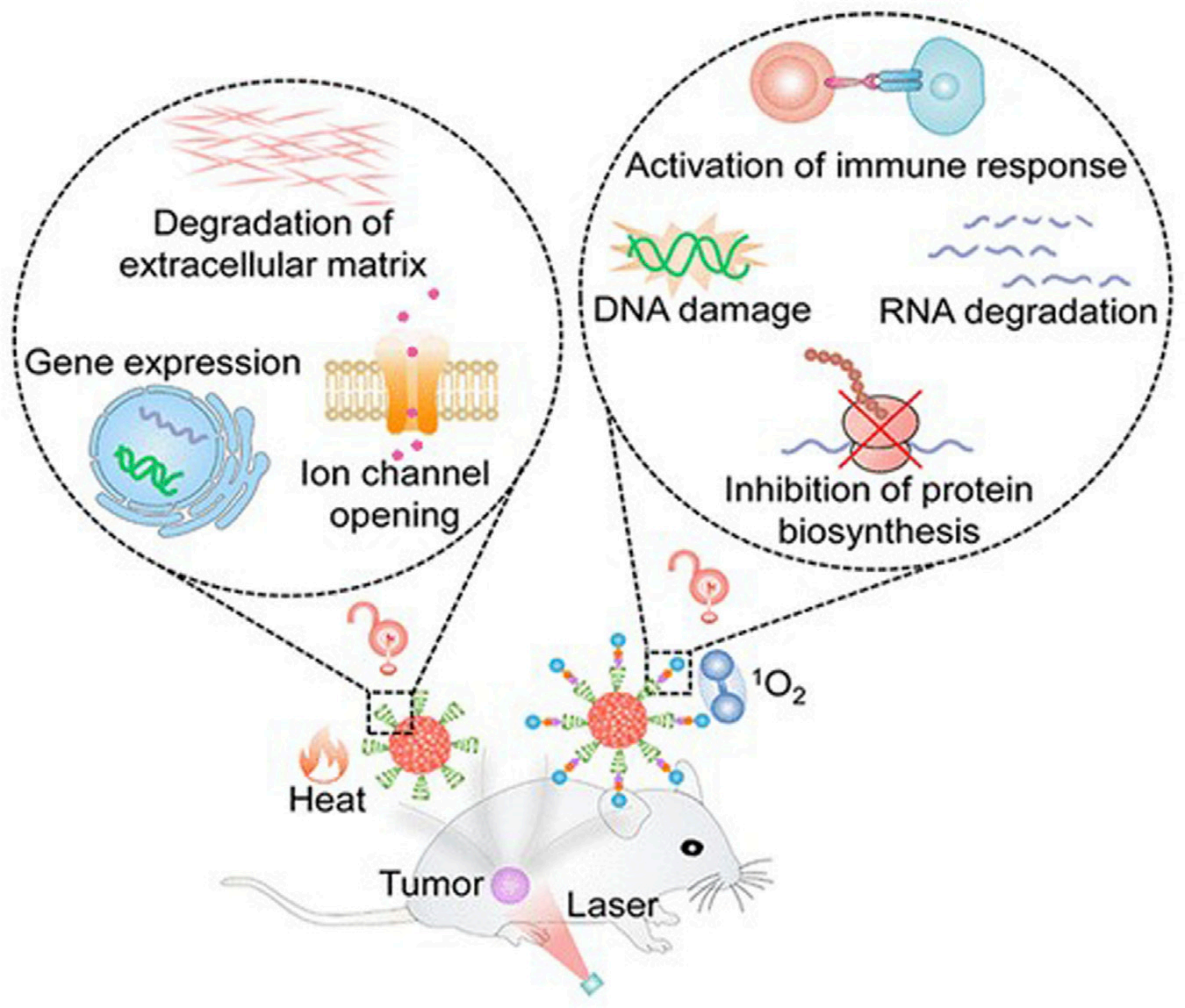
π-conjugated nanomaterials as near-infrared photoactivatable pro-therapeutics for cancer (Cevik et al., 2019).

Photodynamic therapy involves using π-conjugated materials as photosensitizers, exciting their photosensitive properties to generate harmful substances like reactive oxygen or reactive nitrogen species, achieving destruction of brain tumor cells (Lin et al., 2022). Photodynamic therapy offers high selectivity and local efficacy, minimizing damage to surrounding normal brain tissues (Figure 7). Additionally, through targeted modifications, photodynamic therapy can achieve high selective destruction of brain tumors, enhancing treatment precision (Luo et al., 2023).

Researchers proposed a novel π-conjugated polymer nanoparticle guided by nucleic acid conjugates for loading fluorophores. This nanoparticle exhibits infrared emission absorption capacity and can accurately release the fluorophore inside the cell, achieving effective treatment of gliomas that are challenging to treat directly (Cevik et al., 2019). This nanomaterial demonstrates exceptionally high biocompatibility and can be targeted to tumor cells. The application of π-conjugated materials in photodynamic therapy for brain tumors has broad and significant implications. This research is expected to provide a new and more effective treatment strategy for brain cancer (Tia et al., 2010).

However, the application of photothermal therapy and photodynamic therapy also faces some challenges (Cao et al., 2022). For instance, the depth of light penetration limits the therapeutic efficacy for deep-seated brain tumors. Therefore, further optimization of light parameters and the selection of suitable light sources to enhance light penetration depth are necessary. Additionally, research on the metabolic pathways and biodistribution of π-conjugated materials in the body, along with the assessment of the biocompatibility and safety of photothermal therapy and photodynamic therapy, are crucial (Li and Pu, 2020).

### 3.3 Application of π-conjugated materials in combination therapy

Combination therapy is an approach that integrates different treatment strategies to enhance treatment effectiveness. In brain tumor therapy, π-conjugated materials, as multifunctional carriers, offer new possibilities for combination therapy (Jiang et al., 2019).

Firstly, π-conjugated materials can be used as nanomedicine carriers to achieve combined drug delivery (Han et al., 2021). By modifying different anti-tumor drugs on the surface of π-conjugated materials, they can be simultaneously delivered to brain tumor tissues, enabling synergistic combination therapy. This approach overcomes the limitations of single-drug treatment and enhances therapeutic efficacy and anti-tumor effects. Additionally, combination therapy can reduce the occurrence of drug resistance and prolong the duration of treatment effectiveness. Research teams have prepared a conjugated material capable of carrying both chemotherapeutic drugs and photosensitizers, introducing them into brain tumor cells (Chowdhury et al., 2022). *In vitro* experiments showed that the conjugated material effectively released drugs into tumor cells and released photosensitizers under light conditions, further destroying tumor cells. Furthermore, experimental results indicated that the conjugated material, after surface modification, can achieve active targeting of tumor cells, enhancing treatment effectiveness. Secondly, the application of π-conjugated materials in photothermal therapy and photodynamic therapy also provides new options for combination therapy. By modifying photosensitizers on the surface of π-conjugated materials, photosensitized induction therapy for brain tumors can be achieved (Lin et al., 2022). Simultaneously, photothermal therapy or photodynamic therapy can be combined with chemotherapy to achieve multi-dimensional attacks on brain tumors. This combination therapy strategy can improve treatment efficacy and minimize patient damage during the treatment process (Guo et al., 2018). Additionally, π-conjugated materials can be used in combination with radiation therapy. By modifying π-conjugated materials in radiation therapy organs, the radiation dose can be enhanced (Li et al., 2020). The optical properties of π-conjugated materials allow them to absorb and scatter radiation, thereby increasing the radiation dose to the tumor. This combination therapy strategy can improve the effectiveness of radiation therapy and reduce radiation damage to surrounding normal tissues (Grimland et al., 2011; Xie et al., 2019).

However, the application of π-conjugated materials in combination therapy also faces some challenges. Firstly, in-depth research on the interaction between different treatment strategies is required (Chowdhury et al., 2022). There may be interactions between different treatment strategies that require careful adjustment of treatment parameters to achieve the best combination therapy effect. Secondly, the safety and interactions of drugs used in combination therapy must be thoroughly considered to ensure the safety and reliability of the treatment process (Du et al., 2016).

In conclusion, the application of π-conjugated materials in combination therapy for brain tumors provides new options and possibilities for brain tumor treatment. By fully utilizing the multifunctional advantages of π-conjugated materials, nanomedicine delivery, photothermal therapy, photodynamic therapy, and radiation therapy can be combined to improve treatment effectiveness, leading to better treatment outcomes and quality of life for brain tumor patients (Zhao et al., 2020). In the future, further research on the interactions and biocompatibility of π-conjugated materials in combination therapy is needed, continuously optimizing treatment strategies to achieve personalized and precise brain tumor treatment.

## 4 Biocompatibility and toxicity assessment of π-conjugated materials in brain tumor therapy

The potential application of π-conjugated materials in brain tumor therapy is promising, but their biocompatibility and toxicity assessment are critical research directions (Xu et al., 2015; Wang et al., 2020). In this section, we will focus on the biocompatibility and toxicity assessment of π-conjugated materials in brain tumor therapy, covering *in vivo* metabolism and distribution, biocompatibility assessment, and considerations for potential toxicity and safety.

### 4.1 *In Vivo* metabolism and distribution

Understanding the *in vivo* metabolism and distribution of π-conjugated materials is crucial for evaluating their biocompatibility (Bai et al., 2020). The *in vivo* metabolism of π-conjugated materials is typically determined by their physical and chemical properties. Nanoscale π-conjugated materials may be cleared through phagocytic cells in the liver and spleen. Additionally, the unique structure of brain vessels and the blood-brain barrier may affect the distribution of π-conjugated materials in brain tissue (Liu et al., 2020).

Research has shown that factors such as the size, shape, surface modification, and encapsulating materials of π-conjugated materials significantly influence their *in vivo* metabolism and distribution. By rationally designing and improving the physical properties of nanoprobes, optimization of *in vivo* metabolism and distribution can be achieved, enhancing the stability and circulation time of nanoprobes (Yoshizawa, 2012; Xu et al., 2020).

### 4.2 Biocompatibility assessment

Comprehensive assessment of the biocompatibility of π-conjugated materials is essential for their application in brain tumor therapy. Biocompatibility assessment aims to understand the interactions and influences between nanoprobes and organisms, predicting their *in vivo* behavior (Zhou et al., 2020). Common biocompatibility assessments include cytotoxicity experiments, *in vitro* blood stability, *in vivo* tissue stimulation, and immunogenicity testing (Xu et al., 2015).

Through *in vitro* and *in vivo* experiments, researchers can assess the toxicity and damage of π-conjugated materials to cells, further understanding their tolerance to organisms. Additionally, evaluations of blood stability can reveal the behavior of nanoprobes in the blood, such as platelet and plasma protein adsorption. These assessments help predict the *in vivo* circulation stability and biodegradability of nanoprobes (Wang et al., 2020).

### 4.3 Considerations for potential toxicity and safety

In the application of π-conjugated materials, potential toxicity and safety issues must be fully considered. Although π-conjugated materials show many potential advantages in brain tumor therapy, their toxicity may lead to adverse effects on normal tissues. Comprehensive evaluation of the potential toxicity and safety of π-conjugated materials requires considering various factors such as dose-dependent toxicity, potential long-term cumulative effects, biocompatibility, and biodegradability (Xie et al., 2019). Additionally, when combining nanoprobes with other treatment modalities, the comprehensive impact on treatment efficacy and toxicity needs to be evaluated (Xu et al., 2015). To ensure the safety and reliability of π-conjugated materials in brain tumor therapy, researchers need to thoroughly understand their biological behavior and interaction mechanisms (Xiang et al., 2020). Furthermore, strict adherence to relevant biosafety evaluation standards and regulations is essential to ensure the safety of nanoprobes in clinical applications (Coelho et al., 2017).

In conclusion, the biocompatibility and toxicity assessment of π-conjugated materials in brain tumor therapy are necessary steps for their clinical application. By thoroughly understanding the *in vivo* metabolism and distribution of nanoprobes, conducting comprehensive biocompatibility evaluations, and considering potential toxicity and safety issues, important references and guarantees can be provided for the rational application of π-conjugated materials in brain tumor therapy. Additionally, data from preclinical experiments and clinical trials will further verify their safety and effectiveness (de Deus et al., 2021).

## 5 Future development and prospects of π-conjugated materials

### 5.1 Development trends and prospective applications

Significant progress has been made in the research of π-conjugated materials in brain tumor therapy, but their future development trends and prospects are still full of challenges and potential (Zhou and Zhan, 2018). With the continuous advancement of nanotechnology and a deeper understanding of the properties of π-conjugated materials, it is expected that more novel nanoprobes will emerge, leading to breakthroughs in brain tumor therapy (Richard et al., 2020).

1. Development of Multifunctional Nanoprobes: One of the future development trends of π-conjugated materials is the construction of more functional nanoprobes. For example, integrating diagnostic and therapeutic functions into one nanoprobe to achieve multimodal imaging and combination therapy, thereby enhancing the accuracy and effectiveness of brain tumor therapy. Multifunctional nanoprobes can also realize multiple-targeted therapy for brain tumors, becoming a crucial strategy for personalized treatment (Yin et al., 2017).
2. Optimization of Targeting Strategies: Future research will focus on optimizing targeting strategies to address the unique microenvironment of brain tumors, such as the blood-brain barrier and high surface expression of tumor cells. By designing appropriate targeting ligands, high-selectivity recognition and treatment of brain tumor tissues can be achieved. Optimizing targeting strategies will help improve the therapeutic efficacy and biocompatibility of nanoprobes (Bu et al., 2022).
3. Achieving Personalized Treatment: Future development will pay more attention to personalized brain tumor therapy. By combining patients’ genomic information and pathological characteristics, customized π-conjugated nanoprobes can be designed for individualized brain tumor treatment. Personalized treatment will maximize treatment effectiveness and reduce patient side effects (Torres et al., 2020; Li X. et al., 2022).
4. Novel Strategies for Combination Therapy: As multifunctional carriers, π-conjugated materials are expected to become a new strategy for combination therapy. Future research will explore the combination of π-conjugated materials with photothermal therapy, immunotherapy, chemotherapy, and other treatment modalities to achieve synergistic enhancement of treatment effects. This comprehensive approach is likely to become the future direction of brain tumor therapy (Chowdhury et al., 2022).

### 5.2 Challenges and solutions in clinical applications

The clinical application of π-conjugated materials in brain tumor therapy faces challenges that warrant closer examination and innovative solutions (Tian et al., 2022).

1. Conversion Efficiency and Stability: In the application of nanoprobes, conversion efficiency and stability are of paramount importance. While we acknowledge the significance of these aspects, it is essential to delve deeper into the associated challenges. Future research should concentrate on enhancing the synthesis methods of π-conjugated materials, optimizing their optical and photothermal properties to boost conversion efficiency and stability. Additionally, a rational design of surface modification of nanoprobes to increase their circulation time is crucial for improving their *in vivo* stability, and this area requires further exploration (Rakstys et al., 2019; Deng et al., 2021).
2. Biocompatibility and Toxicity Assessment: Comprehensive assessment of the biocompatibility and toxicity of π-conjugated materials is pivotal before contemplating clinical applications. This issue merits further attention. Future research should intensify the study of the biocompatibility and toxicity of nanoprobes to ensure their safety and reliability in brain tumor therapy. Furthermore, rigorous adherence to pertinent biosafety evaluation standards and regulations, alongside thorough preclinical evaluations of nanoprobes, is indispensable for mitigating potential risks (Narayanan et al., 2020; Wang et al., 2020).
3. Overcoming the Blood-Brain Barrier: The blood-brain barrier presents a critical challenge in drug delivery to brain tissue. The overcoming of this obstacle requires deeper investigation. Future research should delve into the utilization of nanotechnology and targeting strategies to surmount the blood-brain barrier, enabling efficient delivery of nanoprobes to brain tumor tissues (Fu et al., 2019; Li et al., 2021b).
4. Clinical Validation and Regulation: Achieving the clinical application of π-conjugated materials in brain tumor therapy demands more comprehensive attention to the challenges associated with large-scale clinical studies and regulatory approvals. Future research should emphasize the feasibility and effectiveness of clinical validation, ensuring that the application of π-conjugated materials meets stringent safety and efficacy requirements (Xu et al., 2020; Wu et al., 2021).

In conclusion, π-conjugated materials have vast future development and application prospects in brain tumor therapy. Through the development of multifunctional nanoprobes, optimization of targeting strategies, realization of personalized treatment, exploration of novel strategies for combination therapy, and addressing challenges in clinical applications, π-conjugated materials are expected to bring revolutionary advancements to brain tumor therapy.

In the future, with the continuous advancement of nanotechnology, the synthesis methods of π-conjugated materials will become more refined and controllable. Novel π-conjugated nanoprobes may possess superior optical and photothermal properties, enabling precise imaging and efficient treatment of brain tumors (Barman et al., 2020). The development of multifunctional nanoprobes will make brain tumor therapy more comprehensive and personalized, improving treatment efficacy and reducing patient discomfort and side effects (Takahashi, 2021).

Optimization of targeting strategies is the key to achieving precise treatment of brain tumors. Future research will further study the unique microenvironment of brain tumors, design appropriate targeting ligands, and achieve high-selectivity recognition and treatment of brain tumor tissues (Shiraki et al., 2020). Additionally, the combined application of nanotechnology and targeting strategies will help overcome the limitations of the blood-brain barrier and achieve efficient delivery of nanoprobes to brain tumor tissues.

With the deepening of preclinical experiments and clinical validation, the safety and efficacy of π-conjugated materials in brain tumor therapy will be comprehensively evaluated (Guo et al., 2016). Researchers will strengthen the study of the biocompatibility and toxicity of nanoprobes to ensure their safety and reliability in brain tumor therapy. Moreover, adhering to relevant biosafety evaluation standards and regulations and conducting rigorous preclinical evaluations of nanoprobes will help reduce potential risks (Lai et al., 2021).

However, in the clinical application of π-conjugated materials in brain tumor therapy, there are still some challenges. The design and execution of clinical trials are complex and time-consuming processes, requiring careful consideration of factors such as sample size, treatment dosage, and patient selection (Hiroto and Wu, 2019). Additionally, nanotechnology may face cost and industrialization issues in large-scale production and application. To achieve the clinical application of π-conjugated materials in brain tumor therapy, further strengthening multidisciplinary cooperation and promoting the close integration of basic research and clinical practice are needed (Haque et al., 2020).

Overall, π-conjugated materials have vast potential for application in brain tumor imaging and therapy (Oubaha et al., 2019). Through continuous research and innovation, nanotechnology is expected to bring revolutionary changes to brain tumor therapy. In the future, we can expect π-conjugated materials to play a greater role in brain tumor therapy, providing more effective and personalized treatment options for patients.

## 6 Conclusion

### 6.1 Contributions of π-conjugated materials in brain tumor imaging and treatment

π-Conjugated materials have demonstrated substantial potential and made noteworthy contributions to brain tumor imaging and treatment. These multifunctional nanoprobes offer several advantages in brain tumor imaging. Through meticulous design and modifications, nanoprobes can achieve highly selective brain tumor imaging, providing high-resolution and high-contrast images. The realization of multimodal imaging facilitates the acquisition of multiple types of imaging data on a single platform, delivering comprehensive brain tumor information to clinical practitioners, thereby assisting in more precise diagnosis and treatment decisions.

In terms of brain tumor treatment, π-conjugated materials, when employed as drug carriers, offer unique advantages. Nanoscale π-conjugated materials can selectively target brain tumors using targeting strategies, thereby enhancing the precise delivery of therapeutic drugs and reducing toxicity to normal tissues. Additionally, the combined use of optical techniques, such as photothermal therapy and photodynamic therapy, enables the precise treatment of brain tumors, consequently improving treatment efficacy.

### 6.2 Future development prospects

The future development prospects of π-conjugated materials in brain tumor imaging and treatment are highly promising. As nanotechnology and biomedical fields continue to advance, we can anticipate the following developments:

1. Design and Synthesis of Novel Nanoprobes: Future research endeavors will persist in exploring novel methods for designing and synthesizing π-conjugated materials. By enhancing the material’s structure and physicochemical properties, nanoprobes can achieve multifunctionality and heightened efficiency, providing an enhanced platform for brain tumor imaging and treatment.
2. Optimization of Biocompatibility and Safety: Future research will focus on optimizing the biocompatibility and safety evaluation of π-conjugated materials. Enhancing the biocompatibility of nanoprobes and diminishing their toxicity to normal tissues will augment their reliability and safety in clinical applications.
3. Implementation of Personalized Treatment: The future will see the design of personalized π-conjugated nanoprobes that consider patients’ genomic information and pathological characteristics for individualized brain tumor treatment. Personalized treatment will better cater to patients’ specific needs, thereby improving treatment efficacy.
4. Application of Multimodal Imaging and Combination Therapy:*Subsequent research will delve into new strategies for multimodal imaging and combination therapy. By integrating multiple imaging and therapeutic functions into a single nanoprobe, comprehensive brain tumor monitoring and treatment can be achieved, augmenting the efficacy of brain tumor therapy.
5. Promotion of Clinical Application: Following advancements in the application of π-conjugated materials in brain tumor therapy, their clinical utilization will gradually expand. Large-scale clinical studies and monitoring will further validate the safety and efficacy of π-conjugated materials, promoting their clinical application.

In summation, π-conjugated materials exhibit extensive potential and offer promising application prospects in brain tumor imaging and treatment. Future research will concentrate on optimizing nanoprobe design and synthesis, improving biocompatibility and safety, implementing personalized treatment and multimodal combination therapy, and advancing the clinical application of π-conjugated materials. With the continuous advancement of science and technology, π-conjugated materials are poised to provide more precise and effective treatment approaches for brain tumors, ultimately enhancing treatment efficacy and the quality of life for individuals affected by brain tumors.
